# Di-μ-thio­semicarbazide-κ^4^ *S*:*S*-bis­[bis­(thio­semicarbazide-κ*S*)copper(I)] diiodide

**DOI:** 10.1107/S1600536808014001

**Published:** 2008-05-17

**Authors:** Li Jia, Shou-Xin Ma, Da-Cheng Li

**Affiliations:** a Liaocheng Vocational and Technical College, Liaocheng, Shandong 252000, People’s Republic of China; b Shandong Vocational Animal Science and Veterinary College, Weifang, Shandong 261000, People’s Republic of China; c School of Chemistry and Chemical Engineering, Liaocheng University, Shandong 252059, People’s Republic of China

## Abstract

The title compound, [Cu_2_{SC(NH_2_)NHNH_2_}_6_]I_2_, was obtained by the reaction of CuI and thio­semicarbazide (TSCZ) in acetonitrile. Each Cu^I^ ion is coordinated by four S atoms of the TSCZ ligands, forming a tetra­hedral geometry. Centrosymmetric dimers are formed by two coordination tetra­hedra sharing a common edge, with a Cu⋯Cu distance of 2.8236 (14) Å. The I^−^ ion does not have any direct inter­action with the metal. The crystal structure is stabilized by weak N—H⋯N, N—H⋯S and N—H⋯I hydrogen bonds, forming a three-dimensional network structure.

## Related literature

For similar structures, see: Chattopadhyay *et al.* (1991 ▶); Burrows *et al.* (2004 ▶); Tong *et al.* (2000 ▶).
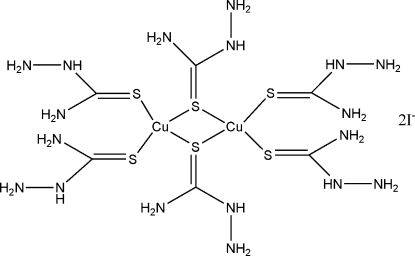


## Experimental

### 

#### Crystal data

[Cu_2_(CH_5_N_3_S)_6_]I_2_
*M* *_r_* = 927.72
Monoclinic, 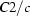

*a* = 16.437 (4) Å
*b* = 8.4174 (15) Å
*c* = 22.546 (4) Å
β = 105.385 (5)°
*V* = 3007.6 (10) Å^3^
*Z* = 4
Mo *K*α radiation
μ = 3.92 mm^−1^
*T* = 273 (2) K
0.45 × 0.37 × 0.23 mm

#### Data collection

Bruker SMART CCD diffractometer
Absorption correction: multi-scan (*SADABS*; Sheldrick, 1996 ▶) *T*_min_ = 0.272, *T*_max_ = 0.466 (expected range = 0.237–0.406)
7573 measured reflections
2636 independent reflections
2135 reflections with *I* > 2σ(*I*)
*R*_int_ = 0.040

#### Refinement

*R*[*F* ^2^ > 2σ(*F* ^2^)] = 0.037
*wR*(*F* ^2^) = 0.106
*S* = 1.01
2636 reflections
154 parameters
H-atom parameters constrained
Δρ_max_ = 0.87 e Å^−3^
Δρ_min_ = −0.99 e Å^−3^

### 

Data collection: *SMART* (Bruker, 1997 ▶); cell refinement: *SAINT* (Bruker, 1997 ▶); data reduction: *SAINT*; program(s) used to solve structure: *SHELXS97* (Sheldrick, 2008 ▶); program(s) used to refine structure: *SHELXL97* (Sheldrick, 2008 ▶); molecular graphics: *SHELXTL* (Sheldrick, 2008 ▶); software used to prepare material for publication: *SHELXTL*.

## Supplementary Material

Crystal structure: contains datablocks I, global. DOI: 10.1107/S1600536808014001/cf2195sup1.cif

Structure factors: contains datablocks I. DOI: 10.1107/S1600536808014001/cf2195Isup2.hkl

Additional supplementary materials:  crystallographic information; 3D view; checkCIF report

## Figures and Tables

**Table 1:** Hydrogen-bond geometry (Å, °)

*D*—H⋯*A*	*D*—H	H⋯*A*	*D*⋯*A*	*D*—H⋯*A*
N1—H1*A*⋯N9^i^	0.86	2.44	3.219 (6)	152
N1—H1*B*⋯S2	0.86	2.73	3.426 (5)	140
N2—H2⋯I1^ii^	0.86	2.80	3.526 (5)	143
N3—H3*B*⋯S3^iii^	0.86	2.95	3.692 (5)	145
N4—H4*A*⋯S2^iv^	0.86	2.72	3.446 (4)	142
N4—H4*B*⋯N6^v^	0.86	2.38	3.225 (6)	167
N5—H5⋯S1	0.86	2.79	3.424 (4)	132
N6—H6*A*⋯N4^iv^	0.86	2.52	3.225 (6)	139
N7—H7*B*⋯I1^vi^	0.86	3.15	3.620 (4)	117
N8—H8⋯S1^vii^	0.86	2.68	3.499 (4)	161
N9—H9*B*⋯I1^viii^	0.86	2.98	3.608 (5)	132

Symmetry codes: (i) 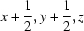; (ii) 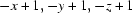; (iii) 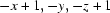; (iv) 
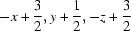
; (v) 
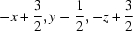
; (vi) 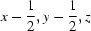; (vii) 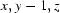; (viii) 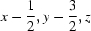.

**Fig. 1.**
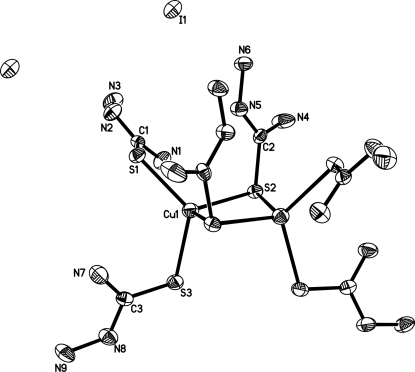
The molecular structure of (I), with atom labels and 40% probability displacement ellipsoids for non-H atoms. [Symmetry code for unlabeled atoms: 1 - x, y, 3/2 - z.]

**Fig. 2.**
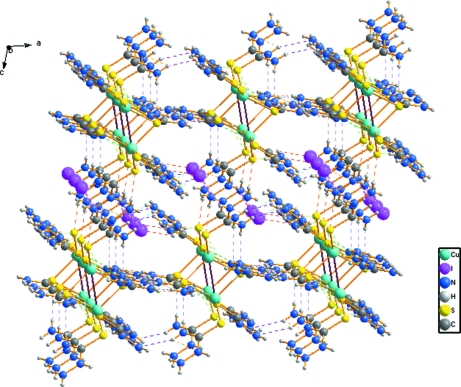
Three-dimensional network structure of the title complex.

## supplementary crystallographic information

### Comment

In previous papers, thiosemicarbazide (TSCZ) has two coordination types; one is as a monodentate S-donor (Chattopadhyay *et al.*, 1991; Tong *et al.*, 2000), the other is as an S,*N*-chelating agent (Burrows *et al.*, 2004). We report the synthesis and the structure of a TSCZ complex of cuprous iodide, (I), in which TSCZ acts as a monodentate S-donor. As shown in Fig. 1, each Cu^I^ atom is in a tetrahedral coordination environment. It is coordinated by two bridging TSCZ ligands and two terminal TSCZ ligands. The Cu—S distances are 2.3118 (14), 2.3192 (13), 2.4098 (13) and 2.4136 (14) Å, which are longer than 2.2266 (1) Å for [Cu(SC(NH_2_)NHNH_2_)Cl_2_] (Chattopadhyay *et al.*, 1991). The bond lengths for S=C are 1.730 (5), 1.726 (4) and 1.702 (5) Å; the corresponding bond length in [Cu(SC(NH_2_)NHNH_2_)Cl_2_] is 1.717 (4)Å (Chattopadhyay *et al.*, 1991).

In the crystal structure, hydrogen bonds are involved. Intramolecular N—H···S interactions appear to influence the conformation of the dimer, while intermolecular N—H···N, N—H···S and N—H···I interactions link the dimers and anions into a three-dimensional network structure (Fig. 2).

### Experimental

CuI (0.19 g 1 mmol) and thiosemicarbazide (0.18 g, 2 mmol) were refluxed in 10 ml acetonitrile for 24 h, and a colorless solution formed. After filtration, the solution was allowed to evaporate slowly, and crystals suitable for X-ray diffraction were obtained after several days.

### Refinement

All H atoms were positioned geometrically and treated as riding on their parent atoms, with N—H = 0.86 Å and *U*_iso_(H) = 1.2*U*_eq_(N).

### Crystal data

**Table:**

[Cu_2_(C_1_H_5_N_3_S_1_)_6_]I_2_	*F*_000_ = 1808
*M**_r_* = 927.72	*D*_x_ = 2.049 Mg m^−^^3^
Monoclinic, *C*2/*c*	Mo *K*α radiation λ = 0.71073 Å
*a* = 16.437 (4) Å	Cell parameters from 4159 reflections
*b* = 8.4174 (15) Å	θ = 2.6–27.8º
*c* = 22.546 (4) Å	µ = 3.92 mm^−^^1^
β = 105.385 (5)º	*T* = 273 (2) K
*V* = 3007.6 (10) Å^3^	Block, colorless
*Z* = 4	0.45 × 0.37 × 0.23 mm

### Data collection

**Table:**

Bruker SMART CCD diffractometer	2636 independent reflections
Radiation source: fine-focus sealed tube	2135 reflections with *I* > 2σ(*I*)
Monochromator: graphite	*R*_int_ = 0.040
*T* = 273(2) K	θ_max_ = 25.0º
φ and ω scans	θ_min_ = 2.7º
Absorption correction: multi-scan(SADABS; Sheldrick, 1996)	*h* = −19→19
*T*_min_ = 0.272, *T*_max_ = 0.466	*k* = −10→9
7573 measured reflections	*l* = −26→14

### Refinement

**Table:**

Refinement on *F*^2^	Secondary atom site location: difference Fourier map
Least-squares matrix: full	Hydrogen site location: inferred from neighbouring sites
*R*[*F*^2^ > 2σ(*F*^2^)] = 0.037	H-atom parameters constrained
*wR*(*F*^2^) = 0.106	*w* = 1/[σ^2^(*F*_o_^2^) + (0.059*P*)^2^ + 7.7455*P*] where *P* = (*F*_o_^2^ + 2*F*_c_^2^)/3
*S* = 1.01	(Δ/σ)_max_ < 0.001
2636 reflections	Δρ_max_ = 0.87 e Å^−^^3^
154 parameters	Δρ_min_ = −0.99 e Å^−^^3^
Primary atom site location: structure-invariant direct methods	Extinction correction: none

### Special details

**Table:**

Geometry. All e.s.d.'s (except the e.s.d. in the dihedral angle between two l.s. planes) are estimated using the full covariance matrix. The cell e.s.d.'s are taken into account individually in the estimation of e.s.d.'s in distances, angles and torsion angles; correlations between e.s.d.'s in cell parameters are only used when they are defined by crystal symmetry. An approximate (isotropic) treatment of cell e.s.d.'s is used for estimating e.s.d.'s involving l.s. planes.
Refinement. Refinement of *F*^2^ against ALL reflections. The weighted *R*-factor *wR* and goodness of fit *S* are based on *F*^2^, conventional *R*-factors *R* are based on *F*, with *F* set to zero for negative *F*^2^. The threshold expression of *F*^2^ > σ(*F*^2^) is used only for calculating *R*-factors(gt) *etc*. and is not relevant to the choice of reflections for refinement. *R*-factors based on *F*^2^ are statistically about twice as large as those based on *F*, and *R*- factors based on ALL data will be even larger.

### Fractional atomic coordinates and isotropic or equivalent isotropic displacement parameters (Å^2^)

**Table:**

	*x*	*y*	*z*	*U*_iso_*/*U*_eq_	
Cu1	0.46872 (4)	−0.07773 (6)	0.68532 (3)	0.04027 (19)	
I1	0.65686 (2)	0.58550 (4)	0.582631 (19)	0.05668 (17)	
N1	0.5760 (3)	0.0278 (5)	0.5883 (2)	0.0488 (11)	
H1A	0.6145	0.0354	0.5689	0.059*	
H1B	0.5809	−0.0413	0.6171	0.059*	
N2	0.5046 (3)	0.2253 (5)	0.5297 (2)	0.0543 (12)	
H2	0.4618	0.2879	0.5195	0.065*	
N3	0.5685 (3)	0.2350 (6)	0.4990 (2)	0.0617 (13)	
H3A	0.6114	0.1726	0.5091	0.074*	
H3B	0.5647	0.3034	0.4700	0.074*	
N4	0.7280 (3)	0.1713 (5)	0.7543 (3)	0.0783 (19)	
H4A	0.7449	0.2677	0.7531	0.094*	
H4B	0.7643	0.0957	0.7643	0.094*	
N5	0.5934 (2)	0.2573 (4)	0.7257 (2)	0.0407 (9)	
H5	0.5402	0.2375	0.7164	0.049*	
N6	0.6214 (3)	0.4144 (4)	0.7244 (2)	0.0467 (11)	
H6A	0.6746	0.4347	0.7336	0.056*	
H6B	0.5854	0.4904	0.7144	0.056*	
N7	0.2933 (3)	−0.2581 (5)	0.5981 (2)	0.0601 (14)	
H7A	0.2449	−0.2781	0.5732	0.072*	
H7B	0.3049	−0.1636	0.6124	0.072*	
N8	0.3286 (3)	−0.5151 (5)	0.5913 (2)	0.0492 (11)	
H8	0.3644	−0.5916	0.6010	0.059*	
N9	0.2482 (3)	−0.5449 (5)	0.5508 (2)	0.0555 (12)	
H9A	0.2120	−0.4692	0.5409	0.067*	
H9B	0.2355	−0.6387	0.5362	0.067*	
S1	0.42975 (7)	0.11539 (13)	0.61021 (6)	0.0390 (3)	
S2	0.61502 (7)	−0.05460 (13)	0.74208 (6)	0.0344 (3)	
S3	0.44805 (8)	−0.34566 (13)	0.66223 (6)	0.0423 (3)	
C1	0.5100 (3)	0.1217 (5)	0.5738 (2)	0.0361 (10)	
C2	0.6478 (3)	0.1399 (5)	0.7409 (2)	0.0347 (10)	
C3	0.3498 (3)	−0.3726 (5)	0.6145 (2)	0.0360 (11)	

### Atomic displacement parameters (Å^2^)

**Table:**

	*U*^11^	*U*^22^	*U*^33^	*U*^12^	*U*^13^	*U*^23^
Cu1	0.0445 (4)	0.0355 (3)	0.0419 (4)	−0.0033 (2)	0.0134 (3)	0.0014 (3)
I1	0.0626 (3)	0.0556 (3)	0.0537 (3)	0.01216 (16)	0.0185 (2)	0.01200 (17)
N1	0.049 (3)	0.056 (2)	0.047 (3)	0.011 (2)	0.023 (2)	0.011 (2)
N2	0.065 (3)	0.052 (3)	0.049 (3)	0.006 (2)	0.020 (2)	0.017 (2)
N3	0.081 (3)	0.063 (3)	0.051 (3)	0.006 (2)	0.035 (3)	0.013 (2)
N4	0.032 (3)	0.043 (3)	0.143 (6)	−0.0066 (19)	−0.006 (3)	0.028 (3)
N5	0.030 (2)	0.0341 (19)	0.057 (3)	−0.0014 (16)	0.0111 (18)	−0.0001 (19)
N6	0.035 (2)	0.032 (2)	0.069 (3)	−0.0013 (15)	0.006 (2)	0.0072 (19)
N7	0.044 (3)	0.041 (2)	0.084 (4)	0.008 (2)	−0.004 (2)	−0.010 (2)
N8	0.046 (2)	0.035 (2)	0.066 (3)	0.0022 (18)	0.013 (2)	−0.010 (2)
N9	0.046 (3)	0.045 (2)	0.073 (4)	−0.0051 (19)	0.010 (2)	−0.020 (2)
S1	0.0362 (6)	0.0352 (6)	0.0458 (8)	0.0042 (5)	0.0110 (5)	0.0067 (5)
S2	0.0296 (6)	0.0322 (5)	0.0427 (7)	0.0013 (4)	0.0118 (5)	0.0042 (5)
S3	0.0418 (7)	0.0307 (6)	0.0511 (8)	0.0054 (5)	0.0064 (6)	−0.0008 (5)
C1	0.044 (3)	0.028 (2)	0.034 (3)	−0.0051 (19)	0.006 (2)	−0.001 (2)
C2	0.031 (2)	0.038 (2)	0.034 (3)	−0.0014 (19)	0.007 (2)	0.009 (2)
C3	0.040 (3)	0.032 (2)	0.041 (3)	−0.0030 (19)	0.019 (2)	−0.002 (2)

### Geometric parameters (Å, °)

**Table:**

Cu1—S1	2.3118 (14)	N5—N6	1.404 (5)
Cu1—S3	2.3192 (13)	N5—H5	0.860
Cu1—S2^i^	2.4098 (13)	N6—H6A	0.860
Cu1—S2	2.4136 (14)	N6—H6B	0.860
Cu1—Cu1^i^	2.8236 (14)	N7—C3	1.321 (6)
N1—C1	1.312 (6)	N7—H7A	0.860
N1—H1A	0.860	N7—H7B	0.860
N1—H1B	0.860	N8—C3	1.318 (6)
N2—C1	1.307 (6)	N8—N9	1.415 (6)
N2—N3	1.405 (6)	N8—H8	0.860
N2—H2	0.860	N9—H9A	0.860
N3—H3A	0.860	N9—H9B	0.860
N3—H3B	0.860	S1—C1	1.730 (5)
N4—C2	1.300 (6)	S2—C2	1.726 (4)
N4—H4A	0.860	S2—Cu1^i^	2.4098 (13)
N4—H4B	0.860	S3—C3	1.702 (5)
N5—C2	1.315 (6)		
			
S1—Cu1—S3	121.59 (6)	N5—N6—H6B	120.0
S1—Cu1—S2^i^	110.09 (5)	H6A—N6—H6B	120.0
S3—Cu1—S2^i^	98.91 (5)	C3—N7—H7A	120.0
S1—Cu1—S2	112.03 (5)	C3—N7—H7B	120.0
S3—Cu1—S2	105.28 (5)	H7A—N7—H7B	120.0
S2^i^—Cu1—S2	107.55 (4)	C3—N8—N9	121.4 (4)
S1—Cu1—Cu1^i^	135.30 (4)	C3—N8—H8	119.3
S3—Cu1—Cu1^i^	102.91 (4)	N9—N8—H8	119.3
S2^i^—Cu1—Cu1^i^	54.23 (4)	N8—N9—H9A	120.0
S2—Cu1—Cu1^i^	54.11 (4)	N8—N9—H9B	120.0
C1—N1—H1A	120.0	H9A—N9—H9B	120.0
C1—N1—H1B	120.0	C1—S1—Cu1	105.77 (16)
H1A—N1—H1B	120.0	C2—S2—Cu1^i^	108.92 (16)
C1—N2—N3	120.4 (4)	C2—S2—Cu1	109.82 (16)
C1—N2—H2	119.8	Cu1^i^—S2—Cu1	71.66 (4)
N3—N2—H2	119.8	C3—S3—Cu1	109.23 (15)
N2—N3—H3A	120.0	N2—C1—N1	118.4 (5)
N2—N3—H3B	120.0	N2—C1—S1	118.4 (4)
H3A—N3—H3B	120.0	N1—C1—S1	123.2 (4)
C2—N4—H4A	120.0	N4—C2—N5	119.0 (4)
C2—N4—H4B	120.0	N4—C2—S2	119.4 (4)
H4A—N4—H4B	120.0	N5—C2—S2	121.6 (3)
C2—N5—N6	120.6 (4)	N8—C3—N7	117.4 (5)
C2—N5—H5	119.7	N8—C3—S3	118.6 (4)
N6—N5—H5	119.7	N7—C3—S3	124.0 (4)
N5—N6—H6A	120.0		

Symmetry codes: (i) −*x*+1, *y*, −*z*+3/2.

### Hydrogen-bond geometry (Å, °)

**Table:**

*D*—H···*A*	*D*—H	H···*A*	*D*···*A*	*D*—H···*A*
N1—H1A···N9^ii^	0.86	2.44	3.219 (6)	152
N1—H1B···S2	0.86	2.73	3.426 (5)	140
N2—H2···I1^iii^	0.86	2.80	3.526 (5)	143
N3—H3B···S3^iv^	0.86	2.95	3.692 (5)	145
N4—H4A···S2^v^	0.86	2.72	3.446 (4)	142
N4—H4B···N6^vi^	0.86	2.38	3.225 (6)	167
N5—H5···S1	0.86	2.79	3.424 (4)	132
N6—H6A···N4^v^	0.86	2.52	3.225 (6)	139
N7—H7B···I1^vii^	0.86	3.15	3.620 (4)	117
N8—H8···S1^viii^	0.86	2.68	3.499 (4)	161
N9—H9B···I1^ix^	0.86	2.98	3.608 (5)	132

Symmetry codes: (ii) *x*+1/2, *y*+1/2, *z*; (iii) −*x*+1, −*y*+1, −*z*+1; (iv) −*x*+1, −*y*, −*z*+1; (v) −*x*+3/2, *y*+1/2, −*z*+3/2; (vi) −*x*+3/2, *y*−1/2, −*z*+3/2; (vii) *x*−1/2, *y*−1/2, *z*; (viii) *x*, *y*−1, *z*; (ix) *x*−1/2, *y*−3/2, *z*.
